# Development of a Novel Autophagy-Related Prognostic Signature and Nomogram for Hepatocellular Carcinoma

**DOI:** 10.3389/fonc.2020.591356

**Published:** 2020-12-18

**Authors:** Qiongxuan Fang, Hongsong Chen

**Affiliations:** Peking University Hepatology Institute and Beijing Key Laboratory of Hepatitis C and Immunotherapy for Liver Diseases, Peking University People’s Hospital, Beijing, China

**Keywords:** prognosis, nomogram, gene signature, autophagy-related gene, immune-related pathways

## Abstract

**Background:**

Hepatocellular carcinoma (HCC) is the seventh most common malignancy and the second most common cause of cancer-related deaths. Autophagy plays a crucial role in the development and progression of HCC.

**Methods:**

Univariate and Lasso Cox regression analyses were performed to determine a gene model that was optimal for overall survival (OS) prediction. Patients in the GSE14520 and GSE54236 datasets of the Cancer Genome Atlas (TCGA) were divided into the high-risk and low-risk groups according to established ATG models. Univariate and multivariate Cox regression analyses were used to identify risk factors for OS for the purpose of constructing nomograms. Calibration and receiver operating characteristic (ROC) curves were used to evaluate model performance. Real-time PCR was used to validate the effects of the presence or absence of an autophagy inhibitor on gene expression in HepG2 and Huh7 cell lines.

**Results:**

OS in the high-risk group was significantly shorter than that in the low-risk group. Gene set enrichment analysis (GSEA) indicated that the association between the low-risk group and autophagy- as well as immune-related pathways was significant. ULK2, PPP3CC, and NAFTC1 may play vital roles in preventing HCC progression. Furthermore, tumor environment analysis *via* ESTIMATION indicated that the low-risk group was associated with high immune and stromal scores. Based on EPIC prediction, CD8+ T and B cell fractions in the TCGA and GSE54236 datasets were significantly higher in the low-risk group than those in the high-risk group. Finally, based on the results of univariate and multivariate analyses three variables were selected for nomogram development. The calibration plots showed good agreement between nomogram prediction and actual observations. Inhibition of autophagy resulted in the overexpression of genes constituting the gene model in HepG2 and Huh7 cells.

**Conclusions:**

The current study determined the role played by autophagy-related genes (ATGs) in the progression of HCC and constructed a novel nomogram that predicts OS in HCC patients, through a combined analysis of TCGA and gene expression omnibus (GEO) databases.

## Introduction

Autophagy is a conserved intracellular degradation process by which cell components, such as cellular organelles, proteins, and invading microbes, are degraded in lysosomes to provide basic materials and energy for cells. It serves as a double-edged sword in tumorigenesis and metastasis. For instance, autophagy functions as a tumor suppressive process which suppresses inflammation, maintains genome stability, and prevents chronic tissue damage as well as cell injury, especially in the early stages of tumorigenesis (1–4). Inflammation displays a high degree of association with tumor growth and poor prognosis in many cancer types, including Hepatocellular carcinoma (HCC) (5, 6). According to several studies, inhibition of autophagy elevates the inflammatory response, thereby promoting cancer progression (7–9). By contrast, autophagy enables tumor metabolism and growth, promotes the survival of tumor cells under hypoxic conditions as well as tumorigenesis and induces resistance against therapeutic agents (2, 4, 10, 11). Previous studies have indicated that autophagy supports the survival and proliferation of cancer cells and metastasis-related behavior (12). Knocking out the *FIP200* gene, which is essential for autophagy, inhibited the initiation, progression, and metastasis of breast cancer (12). Inhibition of autophagy using HCQ and Lys05 decreased the proliferation and metastasis of melanoma cells (13). Thus, autophagy plays a dual role in cancer development and progression. Modulation of autophagy-based cancer therapy is complicated, and further clarification via extensive research may be required.

HCC is the seventh most common malignancy and the second most common cause of cancer-related deaths (14). Due to the hepatitis virus being endemic to China, over half of the new cases of HCC worldwide occur in China each year (15). The majority of HCC patients are usually diagnosed at an advanced stage and are therefore not eligible for radical surgery. Sorafenib is the first-line drug used for the treatment of advanced HCC (16). It facilitated the prolongation of overall survival (OS) in HCC patients for a range of 7.9 to 10.7 months (17). Sustained treatment with sorafenib has also been shown to trigger drug resistance in HCC cells. Reportedly, the autophagy inducer SAHA, in combination with sorafenib, significantly enhanced the response against HCC, compared to sorafenib alone (18). Interestingly, because autophagy also plays a vital role by promoting tumor growth and inducing resistance to chemotherapy, the use of autophagy inhibitors is considered a promising strategy against cancer therapy. One such autophagy inhibitor, 3-methyladenine (3-MA), improved the anti-HCC response when combined with cisplatin, doxorubicin, and sorafenib treatment (19). Another autophagy inhibitor, chloroquine (CQ), markedly suppressed the growth of HCC cells, when combined with sorafenib (20). However, it is not clear as to how these two roles can be distinguished from each other and how the appropriate autophagy inhibitor can be selected.

The objective of the current study was to determine the role of autophagy-related genes (ATGs) in the progression of HCC, construct a novel nomogram capable of predicting OS in HCC patients and identify the mechanism underlying these processes *via* a combined analysis of data from the TCGA and GEO databases. We confirmed that a combination of six genes functioned as a novel prognostic signature of HCC. A subsequent exploration of prospective signaling pathways indicated that autophagy played a protective role in the low-risk group, compared to that in the high-risk group.

## Materials and Methods 

### Datasets

RNA-seq transcriptome profiling and clinical data of samples, including 374 LIHC and 50 normal control samples, were downloaded from the UCSC database (http://xena.ucsc.edu/). GSE14520 and GSE54236 datasets were obtained from the GEO database (https://www.ncbi.nlm.nih.gov/geo/) for further validation. A total of 232 ATGs were obtained from the Human Autophagy Database (http://www.autophagy.lu/index.html), and 319 ATGs were acquired from the GO_REGULATION_OF_AUTOPHAGY gene set in the Molecular Signatures Database, v6.2 (http://software.broadinstitute.org/gsea/msigdb/). After deleting overlapping genes in the two databases, a total of 487 ATGs were finally constructed (**Supplementary Table S1**).

### Gene Signature Development

ATGs that were differentially expressed between tumor tissues and normal tissues in the TCGA dataset were evaluated, leading to the identification of 60 differentially expressed ATGs (DEAs), based on the following criteria: |logFC |> 1 and adjusted P < 0.05. Gene Ontology (GO) and Kyoto Encyclopedia Genes and Genomes (KEGG) annotation was analyzed using the “clusterProfiler” R package (21). Next, we performed a univariate Cox regression analysis of the 60 DEAs to determine their prognostic value in HCC. A total of 10 genes were significantly associated with OS in the TCGA dataset with a cut-off of p < 0.05. Furthermore, a Lasso Cox regression analysis was performed to establish a prognostic risk signature using the “glmnet” and “survival” tools of the R package. Six genes with their own coefficients were identified. Based on the median risk score, the patients in the TCGA, GSE14520, and GSE54236 datasets were separated into the high-risk and low-risk groups, as follows; the risk score was calculated as follows: risk score = (0.02217 * expression level of ZKSCAN3) + (−0.11476 * expression level of FEZ1) + (−0.09629 * expression level of APOL1) + (−0.02453 * expression level of PRKAG2) + (0.06311 * expression level of CDK5R1) + (−0.01089 * expression level of ADRB2).

### Gene Set Enrichment Analysis (GSEA)

Patients were divided into a high-risk group and a low-risk group according to established ATG (ZKSCAN3, FEZ1, APOL1, PRKAG2, CDK5R1, and ADRB2) models. GSEA 3.0 software was used to perform a GSEA analysis in order to explore the underlying mechanisms. A P value < 0.01 and an FDR (false discovery rate) of q < 0.25 were considered statistically significant.

### Tumor Environment

We used the ESTIMATE online tool (https://bioinformatics.mdanderson.org/estimate/) to predict the ratio of stromal and immune cells in the high-risk and low-risk groups. The EPIC method calculates the infiltration abundance of immune cells in both TCGA and GEO datasets using the “EPIC” package, which estimates cell composition based on complex tissue transcriptome profiling.

### Nomogram Construction

Finally, independent prognostic factors were confirmed via the results of univariate and multivariate analyses. Variables corresponding to P < 0.05 in the univariate and multivariate analyses were selected for nomogram development. Two hundred and sixteen patients in the GSE14520 dataset were randomly divided into a training cohort (n = 152) and a test cohort (n = 64). Performance of the model was evaluated via discrimination and calibration using Harrell's concordance index (C-index). The area under the time-dependent ROC curve (AUC) analysis was used to evaluate the performance of the nomogram in predicting OS.

### RNA Isolation and Quantitative Real-Time PCR

RNA from HepG2 and Huh7 cell lines was prepared using a Trizol kit (Invitrogen, USA), while cDNA was synthesized using cDNA Synthesis Kits (Toyobo, FSQ-301). Quantitative real-time PCR (qRT-PCR) was performed using SYBR Green (Toyobo, QPK, 201) in an ABI7500 thermocycler. The mRNA data were normalized to the amount of GAPDH, using the ΔΔCT method. The results are presented as relative expression with the control set to 1. Amplification reactions were performed in a total volume of 20 μl under the following conditions: 95°C for 1 min, followed by 40 cycles of 95°C for 15 s, 54°C for 15 s, and 72°C for 45 s, followed by 60°C for 15 s. Primers for ZKSCAN3, FEZ1, APOL1, ADRB2, PRKAG2, CDK5R1, and GAPDH are listed in **Table S2**.

### Statistical Analysis

We used the unpaired Student’s t-test to compare differences in gene expression between the two groups using the Prism 5 software. Univariate and multivariate analyses were performed to explore the independent prognostic role of the gene signature using the Cox regression model method in the “survival” R package. Kaplan-Meier survival curves were generated by the Kaplan-Meier method and the log-rank test was used to compare the OS of the two groups. Receiver operating characteristic (ROC) curves were generated to test the predictive value of the established gene signature for OS using the R package, “survivalROC.” Identification of the independent significant variables that were used to develop the nomogram was based on backward stepwise selection *via* the Akaike information criterion (AIC). All statistical analyses were performed using R software v3.6.0 and Prism 5 software.

## Results 

### Identification of 60 Differentially Expressed ATGs

**Figure 1:**
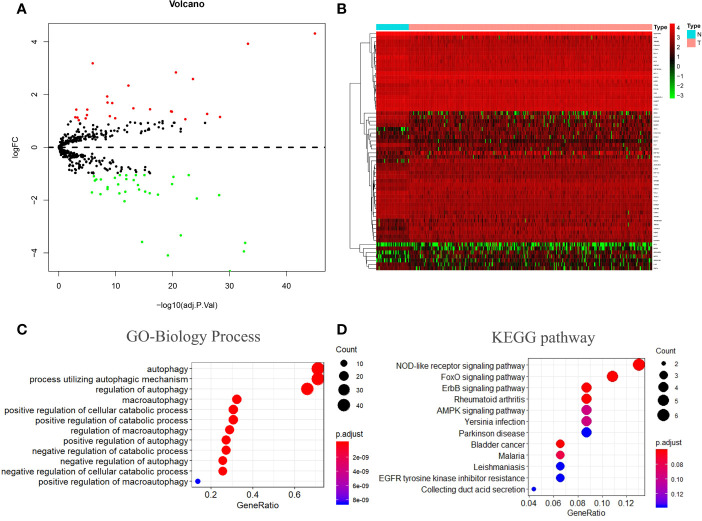
Autophagy-related gene screening and enrichment analysis. **(A, B)** Volcano plot and heatmap of differentially expressed autophagy-related genes in 374 tumor tissues and 50 normal tissues. **(C, D)** Gene ontology (GO) and Kyoto Encyclopedia of Genes and Genomes (KEGG) enrichment analysis. BP stands for biological process, CC stands for cellular component, and MF stands for molecular function.

Of the 487 ATGs, 60 differentially expressed genes (DEAs) were identified in the TCGA dataset based on the following criteria: |logFC |> 1 and an adjusted p value < 0.05. Volcano and heatmap plots are shown (**Figures 1A, B**). Gene ontology (GO) biological process (BP) enrichment analysis of these 60 DEAs (**Table S3**) indicated that these are mainly involved in the regulation of autophagy and catabolic processes (**Figure 1C**). Kyoto Encyclopedia of Genes and Genomes (KEGG) analysis showed that these genes are mainly involved in the Nod-like receptor signaling pathway, the FoxO signaling pathway, and the ErbB signaling pathway (**Figure 1D**).

### Establishment of Prognostic ATG Signature With Six Genes

**Figure 2:**
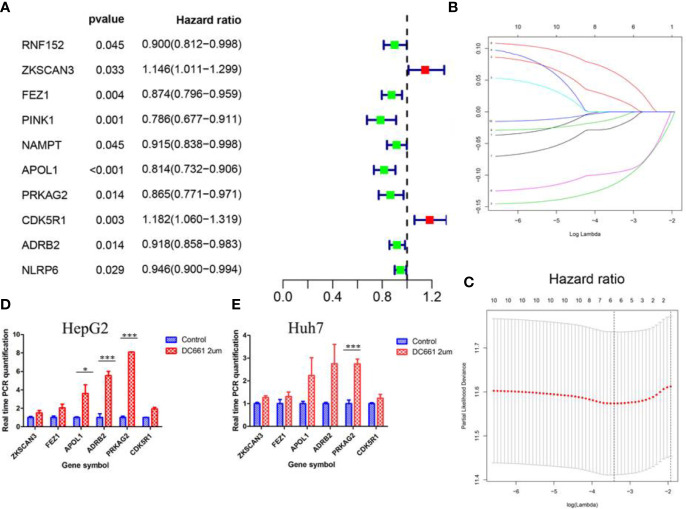
The process of Lasso regression model construction. **(A)** Forest plot of the univariate Cox regression analysis in autophagy-related genes. **(B, C)** Establishment of the Lasso regression model. The expression of the six genes (*FEZ1*, *APOL1*, *PRKAG2*, *ADRB2*, *ZKSCAN3*, and *CDK5R1*) that constituted the model was higher in the autophagy inhibitor (DC661)-treated groups than in the control groups for the HepG2 **(D)** and Huh7 **(E)** cell lines. *p < 0.05; **p < 0.01; ***p < 0.001.

We performed univariate Cox regression analysis to explore the prognostic value of the 60 DEAs identified in HCC patients included in the TCGA dataset. The results showed that 10 genes were significantly correlated with OS (p < 0.05) (**Figure 2A** and **Table 1**). Among the 10 genes, eight were protective genes with a hazard ratio <1, while two were risk genes with a hazard ratio >1 (**Figure 2A**). We then subjected the 10 genes to LASSO Cox regression analysis, and six genes were screened to build the risk signature model (**Figures 2B, C**). The corresponding coefficients and gene expression were used to calculate the risk score, following which the patients in the TCGA and the two GEO datasets were divided into the high- and low-risk groups based on the median risk scores. Among the six identified genes, four (*FEZ1*, *APOL1*, *PRKAG2*, and *ADRB2*) were downregulated, while two (*ZKSCAN3* and *CDK5R1*) were upregulated in the high-risk group.

**Table 1:** The identified 10 significant genes correlated with overall survival.

Gene	Biological and Functional Significance	HR	HR.95L	HR.95H	p-value
RNF152	E3 ubiquitin-protein ligase	0.899807	0.81159	0.99761	0.04492
ZKSCAN3	Zinc finger protein with KRAB and SCAN domains 3	1.146087	1.01089	1.29937	0.03325
FEZ1	Fasciculation and elongation protein zeta-1	0.873598	0.79606	0.9587	0.00438
PINK1	Serine/threonine-protein kinase	0.785582	0.67727	0.91121	0.00143
NAMPT	Nicotinamide phosphoribosyl transferase	0.914729	0.83838	0.99803	0.04504
APOL1	Apolipoprotein L1	0.814163	0.73191	0.90567	0.00016
PRKAG2	5’-AMP-activated protein kinase subunit gamma-2	0.86529	0.77114	0.97093	0.01382
CDK5R1	Cyclin-dependent kinase 5 activator	1.182046	1.05953	1.31873	0.00274
ADRB2	Adrenoceptor beta 2	0.918199	0.85774	0.98292	0.01406
NLRP6	NACHT, LRR, and PYD domains containing protein 6	0.945665	0.89953	0.99417	0.02859

Furthermore, we detected the expression of these six genes in HepG2 and Huh7 cell lines following a 24 h incubation period, in the presence or absence of DC661, a novel dimeric form of CQ that inhibits autophagy (13). The concentration of DC661 (2 μM) was determined based on its half-maximal inhibitory concentration (IC50; data not shown). The expression levels of these six genes were higher in the DC661-treated groups than in the control groups of both cell lines (**Figures 2D, E**). Moreover, the overexpression of *PRKAG2* (p < 0.001) in the DC661-treated groups of both cell lines was significant, while the overexpression of *APOL1* (p < 0.05) and *ADRB2 (*p < 0.01) in the HepG2 cell line was significant.

**Figure 3:**
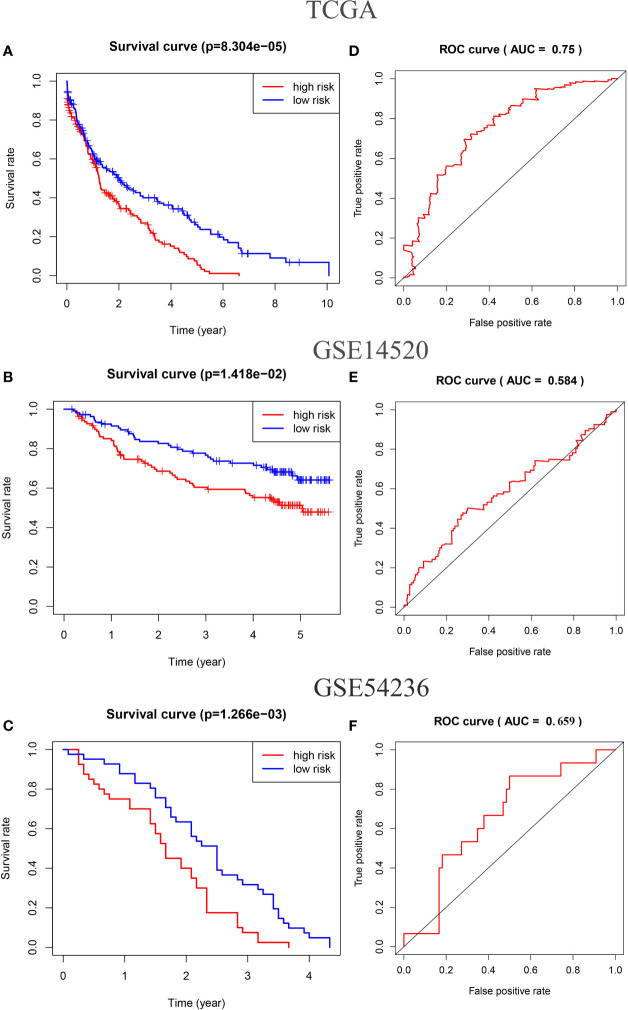
Kaplan–Meier analysis of the high-risk and low-risk groups of HCC samples in the TCGA **(A)**, GSE14520 **(B)**, and GSE54236 datasets **(C)**. The receiver operating characteristic (ROC) curves of the risk score of HCC samples in the TCGA **(D)**, GSE14520 **(E)**, and GSE54236 datasets **(F)**.

Survival analysis showed that OS was shorter in the high-risk group than in the low-risk group (p < 0.01 in TCGA and GSE54236 datasets; p = 0.014 in GSE14520 dataset) (**Figures 3A–C**). The area under the receiver operating characteristic curves (AUCs) for 5-year OS were 0.75 in the TCGA HCC dataset (**Figure 3D**), 0.584 in the GSE14520 dataset (**Figure 3E**), and 0.659 in the GSE54236 dataset (**Figure 3F**).

### Independent Prognostic Role of Gene Signatures in the GSE14520 Cohort

**Figure 4:**
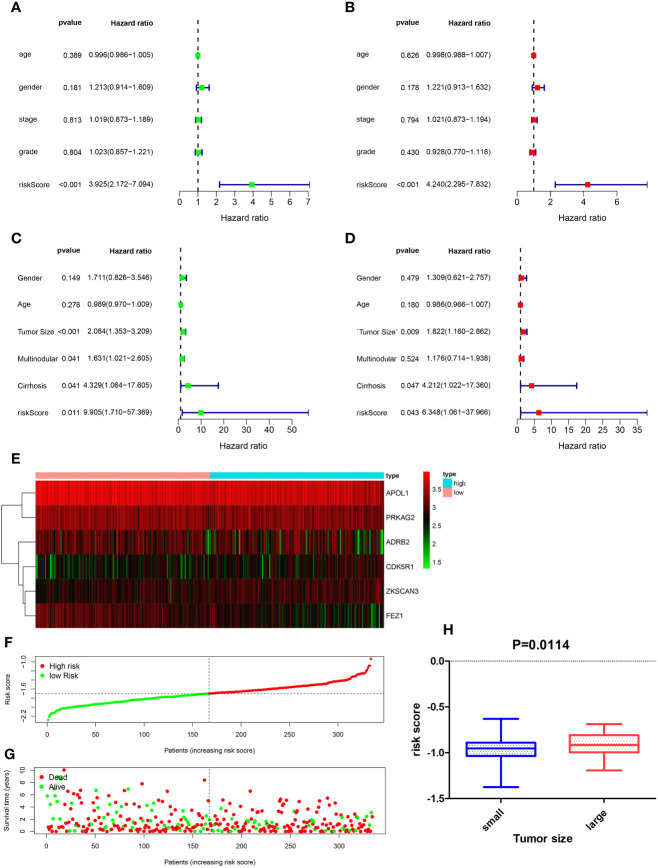
Forest plot of the univariate and multivariate Cox regression analysis in HCC. Univariate regression analysis of HCC samples in the TCGA **(A)** and GSE14520 **(C)** datasets. Multivariate Cox regression analysis of HCC samples in TCGA **(B)** and GSE14520 **(D)** datasets. **(E)** Heatmap of the gene expression profiles of the six autophagy-related genes (ATGs), distribution of prognostic index in the TCGA dataset **(F)**, and survival status of HCC patients in the low- and high-risk groups **(G)**. **(H)** Correlation between tumor size and risk score (p = 0.014).

Univariate and multivariate Cox regression analyses were performed to determine whether ATG signatures played an independent prognostic role in HCC. Both univariate (**Figure 4A**) and multivariate (**Figure 4B**) Cox regression analyses showed that the risk score computed for the TCGA dataset was associated with OS (p < 0.001). A similar result was obtained by validation of the GSE14520 dataset, *via* univariate Cox regression (p = 0.011) and multivariate Cox regression (p = 0.043) analyses (**Figures 4C, D**). Univariate analysis of the GSE14520 cohort indicated that cirrhosis (hazard ratio, 4.329 [95% CI, 1.064–17.605]), risk score (hazard ratio, 9.905 [95% CI, 1.71–57.369]), and tumor size (hazard ratio, 2.084 [95% CI, 1.353–3.209]) were significantly associated with OS, with P-values of 0.041, 0.011, and <0.001, respectively (**Figure 4C**). Multivariate Cox regression analyses indicated that tumor size (hazard ratio, 1.822 [95% CI, 1.16–2.862]), cirrhosis (hazard ratio, 4.212 [95% CI, 1.022–17.36]), and risk score (hazard ratio, 6.348 [95% CI, 1.061–37.966]) were significantly associated with OS, with P-values of 0.009, 0.047, and 0.043, respectively (**Figure 4D**). Moreover, tumor size was significantly correlated with risk score (p = 0.014) (**Figure 4H**). The heatmap of the gene expression profiles of the six ATGs (**Figure 4E**), the distribution of prognostic indices in the TCGA dataset (**Figure 4F**), and the survival status of HCC patients in the low-risk and high-risk groups (**Figure 4G**) are shown. Due to the GSE54236 dataset not containing corresponding clinical information, it was not subjected to univariate and multivariate Cox regression analyses.

### Autophagy-Related Pathways in the Low-Risk Group

**Figure 5:**
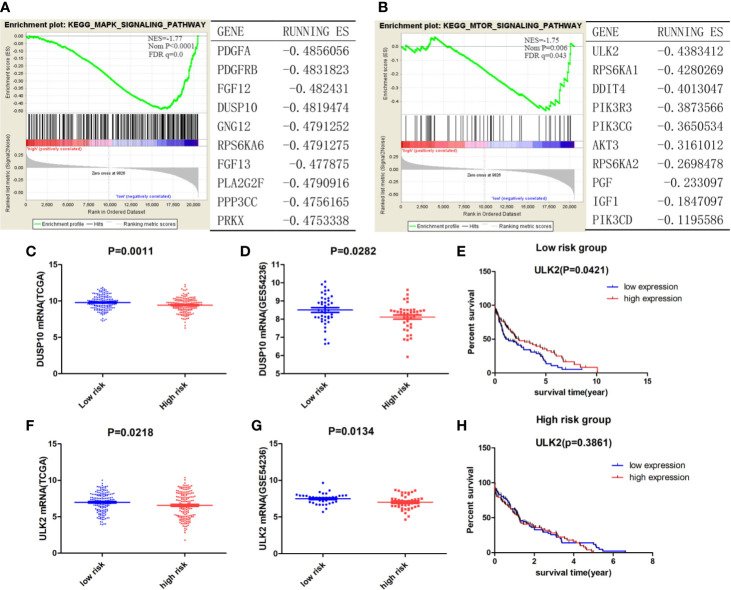
**(A, B)** The enriched autophagy-related Kyoto Encyclopedia of Genes and Genomes (KEGG) pathways and the corresponding top ranked ranking genes in the low-risk group. Genes fulfilling the criteria p < 0.05 and FDR q < 0.25 were considered significant. Expression of DUSP10 in the low- and high-risk groups in both the TCGA **(C)** and GEO **(D)** datasets. Expression of ULK2 between low- and high-risk groups in both the TCGA **(F)** and GEO **(G)** datasets. Survival analysis of patients with high/low ULK2 expression in the low- **(E)** and high-risk **(H)** groups.

As indicated by the GSEA, the low-risk group was significantly associated with autophagy-related pathways (**Figures 5A, B**), such as the MAPK signaling pathway (NES = −1.77, Nom p < 0.001, FDR q = 0.0) and the mTOR signaling pathway (NES = −1.75, Nom p = 0.006, FDR q = 0.043). An analysis of the key genes involved in the above-mentioned autophagy-related pathways revealed that DUSP10 (ES = −0.48), a dual-specificity protein phosphatase (DUSP), negatively regulates members of the mitogen-activated protein kinase (MAPK) superfamily, leading to the indirect activation of autophagy (22) (**Figure 5A**). The low-risk and high-risk groups in both the TCGA and GEO datasets showed significantly different DUSP10 expression levels, but DUSP10 expression in the low-risk group was found to be higher than that in the high-risk group, indicating that the level of autophagy was higher in the low-risk group than in the high-risk group (p = 0.0011 in TCGA dataset, p = 0.0282 in the GSE54236 dataset) (**Figures 5C, D**). ULK2 (ES = −0.44) ranked first in the mTOR signaling pathway; it is well known that ULK1/2 plays a canonical role in inducing autophagy. ULK2 expression in the low-risk group was significantly higher than that in the high-risk group (p = 0.0218 in TCGA dataset; p = 0.0134 in the GSE54236 dataset) (**Figures 5F, G**). Moreover, we categorized HCC patients in the TCGA dataset into the ULK2 high- and low-expression groups based on median expression and survival analysis. In the low-risk group, patients belonging to the high expression group displayed a longer OS than those in the low expression group (p = 0.0421) (**Figure 5E**), whereas there was no significant difference between patients with high and low ULK2 expression in the high-risk group (**Figure 5H**). This indicated that autophagy played a protective role in the low-risk group but not in the high-risk group, and that the MAPK-mTOR pathway may have exerted a significant effect on this process.

### Immune-Related Pathways in the Low-Risk Group

**Figure 6:**
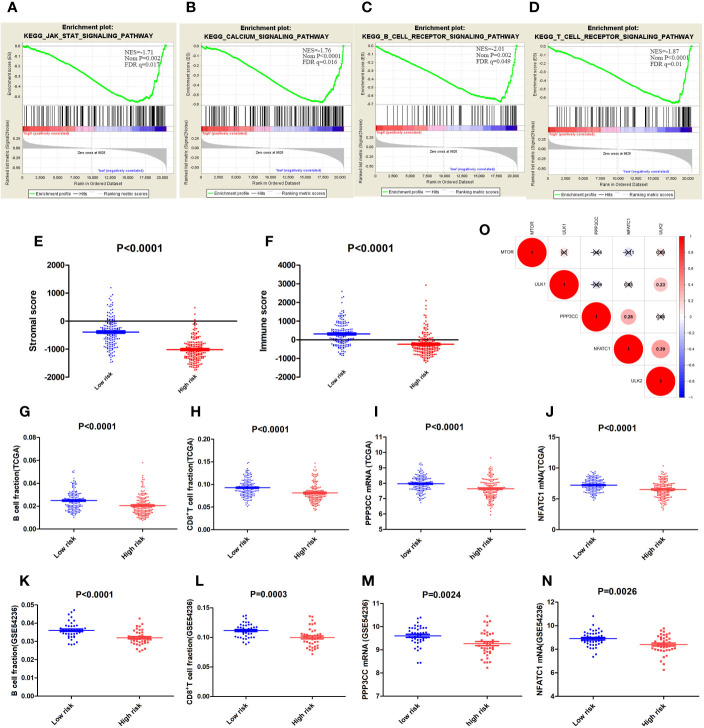
**(A–D)** The enriched immune-related Kyoto Encyclopedia of Genes and Genomes (KEGG) pathways. **(E)** Stromal and immune scores **(F)** in the low- and high-risk groups. B Cell fractions in the low- and high-risk groups in the TCGA **(G)** and GSE54236 **(K)** datasets; and CD8+ T cell fractions in TCGA **(H)** and GSE54236 **(L)** datasets. Expression of PPP3CC in the low- and high-risk groups in TCGA **(I)** and GSE54236 **(M)** datasets; and the expression of NFATC1 in the low- and high-risk groups in TCGA **(J)** and GSE54236 **(N)** datasets. **(O)** Correlation analysis among mTOR, ULK1, ULK2, PPP3CC, and NFATC1. “×” represents p > 0.001.

In addition to autophagy-related pathways, immune-related pathways accounted for a certain percentage in the low-risk group (**Figures 6A–D**); these included the JAK-STAT signaling pathway (NES = −1.71, Nom p = 0.002, FDR q = 0.017), the T cell receptor signaling pathway (NES = −1.87, Nom p < 0.001, FDR q = 0.01), the B cell receptor signaling pathway (NES = −2.01, Nom p =0.002, FDR q = 0.049), and the calcium signaling pathway (NES = −1.76, Nom p < 0.001, FDR q = 0.016). Tumor environment analysis using ESTIMATION showed that the low-risk group was closely associated with high immune and stromal scores (**Figures 6E, F**). EPIC-based prediction of immune cell fractions indicated that B cell and CD8+ T cell fractions in the low-risk group were higher than those in the high-risk group of the TCGA dataset (p < 0.0001 for both B and CD8+ T cells; **Figures 6G, H**) as well as in the GSE54236 dataset (P < 0.0001 for B cells and P = 0.0003 for CD8+T cells; **Figures 6K, L**).

PPP3CC, which ranked ninth in the MAPK signaling pathway (**Figure 5A**), was associated with intracellular Ca^2+^-mediated signals. Moreover, dephosphorylation of PPP3CC may activate NFATC1, a transcription factor that regulates not only activation and proliferation but also the differentiation and programmed death of T-lymphocytes (23); NAFTC1 expression was higher in the low risk group than in the high-risk group. The calcium signaling pathway and the lymphocyte (T and B cell) receptor signaling pathway were also enriched in the low-risk group, indicating that PPP3CC may play a critical role in the regulation of these pathways. The expression of PPP3CC mRNA in the low-risk group was significantly higher than that in the high-risk group (p < 0.0001 in TCGA dataset; p = 0.0024 in the GSE54236 dataset; **Figures 6I, M**). Moreover, the expression of NFATC1 mRNA was also similar (**Figures 6J, N**). Notably, the expression of these three genes, *ULK2*, *PPP3CC*, and *NFATC1*, were significantly and positively correlated; the coefficient of correlation between PPP2CC and NFATC1 was 0.28, while the coefficient of correlation between NFATC1 and ULK2 was 0.39 (p < 0.001) (**Figure 6O**). Our results suggest that based on the ATG signature grouping proposed by us, autophagy may exert a protective effect on the low-risk group by regulating immune system-related pathways.

### Establishment of a Prognostic Nomogram for the OS of HCC Patients

**Figure 7:**
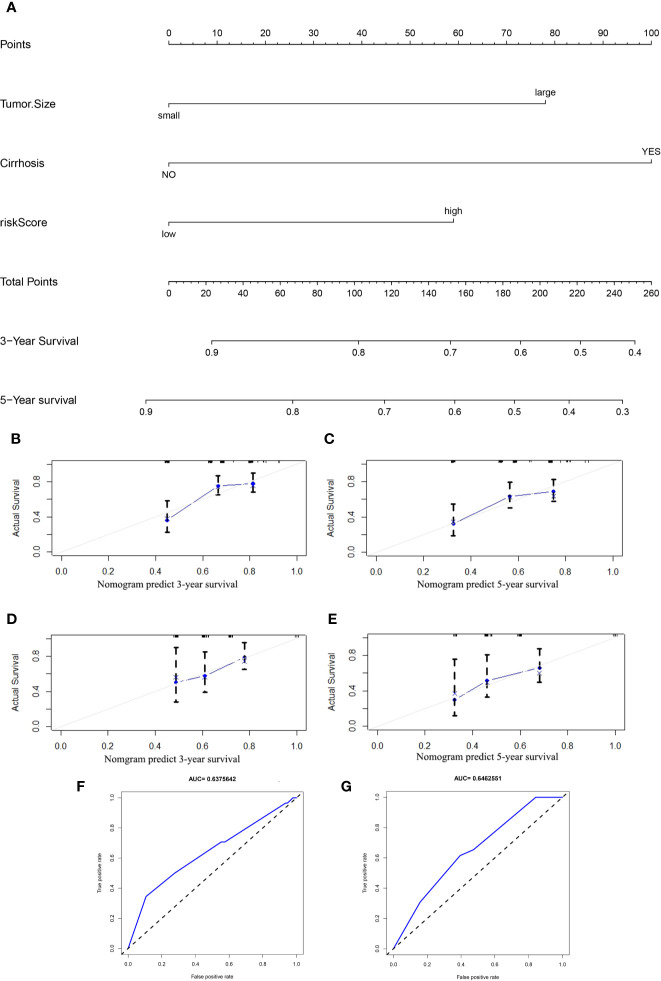
**(A)** Nomogram for predicting the 3- and 5-year OS rates in HCC patients. The calibration curves for predicting the 3- and 5-year OS in the training **(B, C)** and test **(D, E)** cohorts. Receiver operating characteristic (ROC) curves used for predicting the OS in the training **(F)** and test **(G)** cohorts.

Based on the results of the univariate and multivariate analyses (**Figures 4A–D**), clinicopathological characteristics in the GSE14520 dataset were determined to be independent prognostic factors. Univariate analysis of the GSE14520 cohort indicated that cirrhosis (YES or NO), risk score (low or high), and tumor size (small or large), were significantly associated with OS, with P-values of 0.041, 0.011, and < 0.001, respectively (**Figure 4C**). Multivariate Cox regression analysis indicated that tumor size, cirrhosis, and the risk score were significantly associated with OS, as shown by P-values of 0.009, 0.047, and 0.043, respectively (**Figure 4D**). The prognostic nomogram that was generated to predict the OS of HCC patients is shown (**Figure 7A**). The nomogram was used to predict 3- and 5-year OS rates for HCC patients. Each patient is assigned a mortality risk by combining three individual scores identified in the nomogram, where a lower total score is correlated with a better prognosis. According to C statistics, the discriminative ability of the OS prediction model was 0.641 (95% CI, 0.610–0.677) in the training cohort and 0.642 (95% CI, 0.614–0.692) in the test cohort. Prediction of the 3- and 5-year OS rates in the training (**Figures 7B, C**) and test (**Figures 7D, E**) cohorts *via* the bootstrapped calibration plot showed good agreement between nomogram prediction and actual observations. The AUCs of the nomogram score in predicting OS were 0.638 in the training cohort (**Figure 7F**) and 0.646 in the test cohort (**Figure 7G**).

## Discussion 

**Figure 8:**
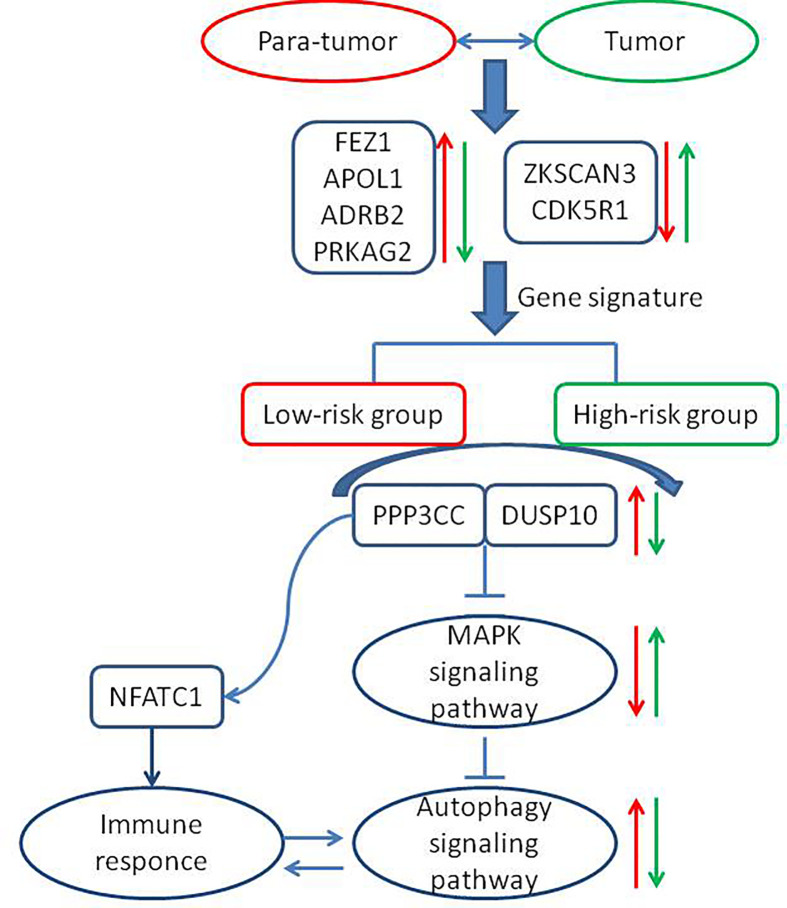
The flow chart depicting the related mechanisms between the newly identified low-risk and high-risk groups. The red arrow indicates the trends in para-tumor tissues or the low-risk group and the green arrow indicates the trends in tumor tissues or the high-risk group. “→” represents increased expression of the corresponding genes. “⊥ “ represents inhibition of the downstream signaling pathways.

The role of autophagy in cancer treatment remains controversial, and whether the induction of autophagy is more advantageous than its inhibition remains unclear (24–26). In the current study, we established a six-ATG signature that categorized HCC patients into the low- and high-risk groups, where a low expression of the gene signature predicted a good prognosis for a patient. The flow chart and the related mechanisms are shown (**Figure 8**). In the HepG2 and Huh7 cell lines, DC661 decreased the extent of autophagy, while increasing the expression of these six genes. Due to inability in linking gene expression to prognosis using liver cancer cell lines, a close link between autophagy and the expression levels of the six identified genes was postulated. Although a previous study has reported using seven ATGs (ATG9A, RAB7A, GNAI3, CAPN10, EIF2S1, SPNS1, and ATG7) to establish prognostic risk signatures for HCC (27), only 232 genes had been included in the Human Autophagy Database. By contrast, our study included a total of 487 ATGs, which are present in both the Human Autophagy and Molecular Signatures databases. Moreover, we constructed a novel nomogram capable of predicting OS for HCC patients, based on identified gene signatures and clinicopathological characteristics, which provide points of reference to clinical physicians. Zhang et al. identified an eight-lncRNA-based nomogram and a 14-lncRNA-based nomogram, which were used to perform the clinicopathological characterization of OS and HCC recurrence, respectively (28). Fu-Biao Ni et al., established a nine-mRNA classifier and used clinicopathologic factors, including age and metastasis, to predict the mortality of HCC patients (29). To our knowledge, our study is the first to generate an ATG signature-based nomogram for predicting OS in HCC. However, a larger multicenter external cohort may be needed for the further validation of the accuracy of this nomogram.

Signaling pathways affecting autophagy, such as the MAPK and mTOR signaling pathways, were enriched in the low-risk group. Autophagy-inducing kinases, such as ULK2, and the MAP kinase negative regulator, DUSP10, were upregulated in the low-risk group, whereas high expression of ULK2 was significantly correlated with better prognoses. These results indicated that the role of autophagy in the low-risk group may be similar to that in normal liver cells, where it removes metabolites, such as excessive protein aggregates, accumulated lipids, and damaged mitochondria (30, 31). Evidently, autophagy also suppresses the growth of tumors by suppressing inflammation (7, 32–34), inhibiting cancer cell growth (26, 35), and regulating the degradation of P62 (36, 37). Many studies have indicated that the inhibition, as well as the induction of autophagy may exert anticancer effects in HCC patients. Therefore, clarification of the role of autophagy at different stages of HCC development may be considered important. The current study helped clarify the role of autophagy in HCC patients classified as low risk.

Reportedly, autophagy occurs not only in cancer cells but also in immune cells (38). Regulation of autophagy in various immune cells may lead to immune cell differentiation and homeostasis (39, 40). The findings of the current study indicated that some immune cell-related signaling pathways mentioned above were also enriched in the low-risk group.

The effect of autophagy on T cells remains unclear. Some findings support the notion that while the induction of autophagy favors the formation and survival of CD8+ T cells, it does not favor either the proliferation or the differentiation of these cells. Moreover, autophagy provides the metabolites that are necessary for the development of memory T cells (41, 42). Other studies indicate that autophagy promotes the apoptosis of T cells (43). Our data revealed that autophagy and immune response may coexist in the low-risk group where key genes, such as *ULK2*, *PPP3CC*, and *NAFTC1*, were significantly and positively correlated. Furthermore, our results demonstrated that PPP3CC may play an essential role in HCC inhibition. *PPP3CC* encodes a regulatory subunit of calcineurin, and PPP3CC dephosphorylation activates the transcription factor NFATC1. The role of PPP3CC in HCC remains unclear. A previous study reported that PPP3CC inhibited cell invasion and growth in glioma (44). A decrease in PPP3CC expression was associated with the recurrence of prostate cancer (45). Jeong et al. further demonstrated that the suppression of the expression of PPP3CC, which is an important member of the signaling circuit (IkBa/NF-kB(p65)/miR-196b-3p/Meis2-PPP3CC), resulted in the phosphorylation of IκBa and activation of NF-kB (p65) in castration-resistant prostate cancer (CRPC) cells. PPP3CC overexpression disorganized the signaling circuit and significantly inhibited the development of CRPC (46). The results of our study indicate that *PPP3CC* plays a beneficial role in HCC development, and that it may trigger the activity of the transcription factor NFATC1, thereby facilitating the activation of T lymphocytes. This hypothesis requires experimental validation.

The current study was beset by certain limitations. First, the established nomogram was not validated using an external dataset, indicating that large, multicenter cohorts are needed to validate the nomogram. Secondly, corresponding *in vitro* and *in vivo* experiments may be needed in order to validate and explore the mechanisms underlying the suppression of HCC by autophagy and immunology.

In the current study, we determined the role of autophagy-related genes (ATGs) in HCC progression and performed a combined analysis of datasets from the TCGA and GEO databases, which enabled the construction of a novel nomogram capable of predicting OS for HCC patients.

## Data Availability Statement

The datasets presented in this study can be found in online repositories. The names of the repository/repositories and accession number(s) can be found in the article/**Supplementary Material**.

## Author Contributions

QF and HC were involved in the study concept and design, analyzing and interpreting the data, and drafting the manuscript. All authors contributed to the article and approved the submitted version.

## Funding

This work was supported by grants from the National Key Sci-Tech Special Project of China (No. 2018ZX10302207 and No. 2017ZX10203202).

## Conflict of Interest

The authors declare that the research was conducted in the absence of any commercial or financial relationships that could be construed as a potential conflict of interest.
