# Supplementary material for: Development of a Novel Autophagy-Related Prognostic Signature and Nomogram for Hepatocellular Carcinoma
Source: Front Oncol. 2020 Dec 18;10:591356. doi: 10.3389/fonc.2020.591356 (PMC7775646; doi:10.3389/fonc.2020.591356)
Supplement: Supplementary file 1 [file Table_1.docx]

Table S1. Autophagy Related genes (ARGs) investigated in this study.

| Gene symbol | Gene symbol | Gene symbol | Gene symbol | Gene symbol |
| --- | --- | --- | --- | --- |
| ABL1 | CISD2 | HSPB1 | NRG3 | SNX32 |
| ABL2 | CLEC16A | HSPB8 | OPTN | SNX5 |
| AC003665.1 | CLN3 | HTR2B | P4HB | SNX6 |
| AC022966.1 | CPTP | HTRA2 | PAFAH1B2 | SOGA1 |
| ACER2 | CSNK2A2 | HTT | PARK2 | SOGA3 |
| ADRA1A | CTSA | IFI16 | PARK7 | SPHK1 |
| ADRB2 | CTSB | IFNG | PARP1 | SPNS1 |
| AKT1 | CTSD | IFT20 | PEA15 | SPTLC1 |
| AMBRA1 | CTSL1 | IFT88 | PELP1 | SPTLC2 |
| APOL1 | CTTN | IKBKB | PEX14 | SQSTM1 |
| ARNT | CX3CL1 | IKBKE | PEX3 | SREBF1 |
| ARSA | CXCR4 | IKBKG | PHF23 | SREBF2 |
| ARSB | DAP | IL10 | PIK3C3 | ST13 |
| ATF4 | DAPK1 | IL10RA | PIK3CA | STAT3 |
| ATF6 | DAPK2 | IL24 | PIK3CB | STK11 |
| ATG10 | DAPK3 | IL4 | PIK3R2 | SUPT5H |
| ATG101 | DAPL1 | IRGM | PIK3R4 | SVIP |
| ATG12 | DCN | ITGA3 | PIKFYVE | TAB2 |
| ATG13 | DDIT3 | ITGA6 | PIM2 | TAB3 |
| ATG16L1 | DEPP1 | ITGB1 | PINK1 | TBC1D14 |
| ATG16L2 | DHRSX | ITGB4 | PIP4K2A | TBC1D25 |
| ATG2A | DIRAS3 | ITPR1 | PIP4K2B | TBK1 |
| ATG2B | DLC1 | KAT8 | PIP4K2C | TFEB |
| ATG3 | DNAJB1 | KDM4A | PLEKHF1 | TICAM1 |
| ATG4A | DNAJB9 | KDR | PLK2 | TIGAR |
| ATG4B | DNM1L | KIAA0226 | PLK3 | TLK2 |
| ATG4C | DNM1P34 | KIAA0652 | POLDIP2 | TM9SF1 |
| ATG4D | DRAM1 | KIAA0831 | PPP1R15A | TMEM150A |
| ATG5 | DRAM2 | KIAA1324 | PRKAA1 | TMEM150B |
| ATG7 | EDEM1 | KIF25 | PRKAA2 | TMEM150C |
| ATG9A | EEF1A1 | KIF5B | PRKAB1 | TMEM49 |
| ATG9B | EEF1A2 | KLHL22 | PRKAB2 | TMEM59 |
| ATIC | EEF2 | KLHL24 | PRKACA | TMEM74 |
| ATM | EEF2K | LACRT | PRKAG1 | TNFSF10 |
| ATP13A2 | EGFR | LAMP1 | PRKAG2 | TOMM7 |
| ATP5IF1 | EIF2AK2 | LAMP2 | PRKAG3 | TP53 |
| ATP6V0A1 | EIF2AK3 | LAMP3 | PRKAR1A | TP53INP1 |
| ATP6V0A2 | EIF2AK4 | LAMTOR1 | PRKCD | TP53INP2 |
| ATP6V0B | EIF2S1 | LAMTOR2 | PRKCQ | TP63 |
| ATP6V0C | EIF4EBP1 | LAMTOR3 | PRKD1 | TP73 |
| ATP6V0D1 | EIF4G1 | LAMTOR4 | PRKN | TPCN1 |
| ATP6V0D2 | EIF4G2 | LAMTOR5 | PSAP | TPCN2 |
| ATP6V0E1 | EP300 | LARP1 | PTEN | TREM2 |
| ATP6V0E2 | EPM2A | LARS | PTK2 | TRIM13 |
| ATP6V1A | ERBB2 | LEP | PTK6 | TRIM21 |
| ATP6V1B1 | ERCC4 | LEPR | PTPN22 | TRIM22 |
| ATP6V1B2 | ERN1 | LRRK2 | PYCARD | TRIM65 |
| ATP6V1C1 | ERO1L | LRSAM1 | QSOX1 | TRIM8 |
| ATP6V1C2 | EXOC1 | LZTS1 | RAB11A | TSC1 |
| ATP6V1D | EXOC4 | MAP1LC3A | RAB12 | TSC2 |
| ATP6V1E1 | EXOC7 | MAP1LC3B | RAB1A | TSPO |
| ATP6V1E2 | EXOC8 | MAP1LC3C | RAB1B | TUSC1 |
| ATP6V1G1 | FADD | MAP2K7 | RAB24 | UBQLN1 |
| ATP6V1G2 | FAM48A | MAP3K7 | RAB33B | UBQLN2 |
| ATP6V1H | FAS | MAPK1 | RAB39B | UBQLN4 |
| BAD | FBXL2 | MAPK15 | RAB3GAP1 | UCHL1 |
| BAG1 | FBXO7 | MAPK3 | RAB3GAP2 | ULK1 |
| BAG3 | FBXW7 | MAPK8 | RAB5A | ULK2 |
| BAK1 | FEZ1 | MAPK8IP1 | RAB7A | ULK3 |
| BAX | FEZ2 | MAPK9 | RAB8A | USP10 |
| BCL2 | FKBP1A | MAPT | RAC1 | USP13 |
| BCL2L1 | FKBP1B | MBTPS2 | RAF1 | USP30 |
| BCL2L11 | FLCN | MCL1 | RALB | USP33 |
| BECN1 | FOS | MEFV | RASIP1 | UVRAG |
| BID | FOXO1 | MET | RB1 | VAMP3 |
| BIRC5 | FOXO3 | MFN2 | RB1CC1 | VAMP7 |
| BIRC6 | FYCO1 | MFSD8 | RELA | VDAC1 |
| BMF | FZD5 | MID2 | RGS19 | VEGFA |
| BNIP1 | GAA | MIR199A1 | RHEB | VPS13C |
| BNIP3 | GABARAP | MIR199A2 | RIPK2 | VPS13D |
| BNIP3L | GABARAPL1 | MIRLET7B | RMC1 | VPS26A |
| BOK | GABARAPL2 | MLST8 | RNF152 | VPS26B |
| C12orf44 | GAPDH | MT3 | RNF41 | VPS35 |
| C17orf88 | GATA4 | MTCL1 | RNF5 | WAC |
| C9orf72 | GBA | MTDH | ROCK1 | WASHC1 |
| CALCOCO2 | GFAP | MTM1 | RPS6KB1 | WDFY3 |
| CAMKK2 | GNAI3 | MTMR14 | RPTOR | WDR24 |
| CANX | GNB2L1 | MTMR3 | RRAGA | WDR41 |
| CAPN1 | GOLGA2 | MTMR8 | RRAGB | WDR45 |
| CAPN10 | GOPC | MTMR9 | RRAGC | WDR45L |
| CAPN2 | GPSM1 | MTOR | RRAGD | WDR6 |
| CAPNS1 | GRID1 | MUL1 | RUBCN | WIPI1 |
| CASP1 | GRID2 | MYC | RUFY4 | WIPI2 |
| CASP3 | GSK3A | NAF1 | SAR1A | XBP1 |
| CASP4 | HAX1 | NAMPT | SCFD1 | XPA |
| CASP8 | HDAC1 | NBR1 | SCOC | ZC3H12A |
| CCL2 | HDAC6 | NCKAP1 | SEC22B | ZFYVE1 |
| CCR2 | HERC1 | NEDD4 | SERPINA1 | ZKSCAN3 |
| CD46 | HGF | NFE2L2 | SESN1 | ZMPSTE24 |
| CDC37 | HGS | NFKB1 | SESN2 | NRG2 |
| CDK5 | HIF1A | NKX2-3 | SESN3 | SNRNP70 |
| CDK5R1 | HK2 | NLRC4 | SH3BP4 |  |
| CDKN1A | HMGB1 | NLRP6 | SH3GLB1 |  |
| CDKN1B | HMGB4 | NOD1 | SIRT1 |  |
| CDKN2A | HMOX1 | NPC1 | SIRT2 |  |
| CFLAR | HS1BP3 | NPRL2 | SLC38A9 |  |
| CHMP2B | HSP90AB1 | NPRL3 | SMCR8 |  |
| CHMP4A | HSPA5 | NRBP2 | SMURF1 |  |
| CHMP4B | HSPA8 | NRG1 | SNCA |  |
