# Supplementary material for: Development of a Novel Autophagy-Related Prognostic Signature and Nomogram for Hepatocellular Carcinoma
Source: Front Oncol. 2020 Dec 18;10:591356. doi: 10.3389/fonc.2020.591356 (PMC7775646; doi:10.3389/fonc.2020.591356)
Supplement: Supplementary file 2 [file Table_2.docx]

| Table S2. Primers used for real time PCR. | |
| --- | --- |
| Gene | Sequences |
| ZKSCAN3 | Forward: 5'-ACCGCGTCATCAAAGCTGT-3" |
|  | Reverse: 5'-CTTCCAGTCGTCCTTCCTTTAC-3' |
| FEZ1 | Forward: 5'-ACTACAACGCCAAGACCGAG-3' |
|  | Reverse:5'-GAGGCTCCTCGTTGATACCG-3' |
| APOL1 | Forward: 5'-CCAATGTGGTGTCTGGCTCT-3' |
|  | Reverse:5'-CCAACTCCATCCCAGGTTCC-3' |
| ADRB2 | Forward: 5'-CGCTTCCATGTCCAGAACCT-3' |
|  | Reverse:5'-GAGGGTGAAAGTGCCCATGA-3' |
| PRKAG2 | Forward: 5'-CCAAAACCGTGTTCCCGTTC-3' |
|  | Reverse:5'-GTGTGCTGCTTGGTCACTTG-3' |
| CDK5R1 | Forward: 5'-GCAAGAACGCCAAGGACAAG-3' |
|  | Reverse: 5'-TTCTGGTAGCTGCTGTTGGG-3' |
| GAPDH | Forward: 5'-GTCTTCACCACCATGGAGAAG-3' |
|  | Reverse:5'-CATGAGTCCTTCCACGATACC-3' |
