# Supplementary material for: Development of a Novel Autophagy-Related Prognostic Signature and Nomogram for Hepatocellular Carcinoma
Source: Front Oncol. 2020 Dec 18;10:591356. doi: 10.3389/fonc.2020.591356 (PMC7775646; doi:10.3389/fonc.2020.591356)
Supplement: Supplementary file 3 [file Table_3.docx]

Table S3. Differentially expressed autophagy related genes.

| Gene | logFC | AveExpr | t | P.Value | adj.P.Val | B |
| --- | --- | --- | --- | --- | --- | --- |
| ADRA1A | -4.6968 | 5.240769 | -12.9975 | 9.57E-33 | 8.78E-31 | 63.55558 |
| DCN | -4.09347 | 9.51585 | -9.96648 | 3.67E-21 | 6.74E-20 | 37.14655 |
| DIRAS3 | -3.94074 | 4.240489 | -13.6233 | 2.60E-35 | 2.98E-33 | 69.4138 |
| FOS | -3.61862 | 10.25518 | -13.707 | 1.17E-35 | 1.79E-33 | 70.20611 |
| NRG1 | -3.58165 | 4.475753 | -8.54375 | 2.36E-16 | 2.36E-15 | 26.22236 |
| HGF | -3.33378 | 5.245435 | -10.6539 | 1.21E-23 | 3.69E-22 | 42.79995 |
| NRG3 | -2.04381 | 2.079171 | -7.48375 | 4.21E-13 | 2.82E-12 | 18.86217 |
| RNF152 | -1.938 | 6.283769 | -11.4512 | 1.22E-26 | 5.61E-25 | 49.62414 |
| FOXO1 | -1.81272 | 9.443616 | -12.493 | 1.03E-30 | 6.74E-29 | 58.92002 |
| FEZ1 | -1.79037 | 6.538696 | -9.439 | 2.53E-19 | 3.74E-18 | 32.96753 |
| LEPR | -1.77645 | 11.14306 | -5.81399 | 1.20E-08 | 4.71E-08 | 8.838208 |
| CCL2 | -1.74167 | 7.893464 | -7.15734 | 3.65E-12 | 2.26E-11 | 16.74461 |
| IL10 | -1.73685 | 2.362822 | -8.99177 | 8.14E-18 | 1.07E-16 | 29.54096 |
| NLRP6 | -1.70663 | 4.699323 | -5.10488 | 5.01E-07 | 1.53E-06 | 5.227656 |
| LRRK2 | -1.68341 | 6.780019 | -7.53526 | 2.97E-13 | 2.04E-12 | 19.20296 |
| HMOX1 | -1.67463 | 10.40571 | -8.72356 | 6.20E-17 | 6.72E-16 | 27.54014 |
| NAMPT | -1.60049 | 11.79569 | -8.36546 | 8.73E-16 | 7.86E-15 | 24.93495 |
| ADRB2 | -1.58167 | 6.969546 | -6.34072 | 5.86E-10 | 2.77E-09 | 11.77772 |
| MYC | -1.5051 | 10.0029 | -6.35123 | 5.51E-10 | 2.66E-09 | 11.83855 |
| GABARAPL1 | -1.42793 | 11.03276 | -8.30105 | 1.39E-15 | 1.23E-14 | 24.47459 |
| SERPINA1 | -1.40209 | 18.89486 | -7.07724 | 6.13E-12 | 3.70E-11 | 16.23623 |
| DLC1 | -1.39199 | 9.682579 | -10.1902 | 5.85E-22 | 1.26E-20 | 38.96121 |
| SNCA | -1.25935 | 2.872184 | -5.37494 | 1.27E-07 | 4.51E-07 | 6.554636 |
| PLK3 | -1.24337 | 7.312306 | -8.04591 | 8.67E-15 | 6.98E-14 | 22.67642 |
| TRIM22 | -1.21877 | 8.801016 | -5.49107 | 6.89E-08 | 2.54E-07 | 7.143584 |
| TUSC1 | -1.21865 | 7.396689 | -5.8444 | 1.01E-08 | 4.02E-08 | 9.002063 |
| APOL1 | -1.20303 | 12.24752 | -7.60188 | 1.89E-13 | 1.34E-12 | 19.64639 |
| MEFV | -1.15361 | 2.950279 | -6.85997 | 2.45E-11 | 1.39E-10 | 14.88005 |
| EPM2A | -1.12192 | 7.054831 | -10.2634 | 3.19E-22 | 7.33E-21 | 39.56087 |
| ACER2 | -1.09568 | 4.044171 | -5.29181 | 1.95E-07 | 6.67E-07 | 6.139757 |
| PRKAG2 | -1.05836 | 9.096979 | -7.5945 | 1.99E-13 | 1.38E-12 | 19.59709 |
| PINK1 | -1.05432 | 11.55182 | -8.6754 | 8.89E-17 | 9.06E-16 | 27.18534 |
| MCL1 | -1.05409 | 13.10882 | -11.0536 | 3.93E-25 | 1.50E-23 | 46.18717 |
| C9orf72 | -1.05371 | 7.544912 | -7.18878 | 2.97E-12 | 1.87E-11 | 16.94536 |
| PPP1R15A | -1.0525 | 9.914387 | -8.17644 | 3.42E-15 | 2.91E-14 | 23.59126 |
| TMEM74 | 1.031257 | 4.729929 | 3.848222 | 0.000137 | 0.000307 | -0.13153 |
| TRIM65 | 1.064166 | 8.428274 | 10.88795 | 1.64E-24 | 5.79E-23 | 44.77493 |
| IKBKE | 1.095314 | 6.697828 | 4.548747 | 7.05E-06 | 1.92E-05 | 2.687372 |
| CDK5R1 | 1.102239 | 5.463594 | 6.884133 | 2.10E-11 | 1.21E-10 | 15.02922 |
| KIAA1324 | 1.123853 | 4.208054 | 3.702047 | 0.000242 | 0.000519 | -0.66464 |
| MT3 | 1.136928 | 1.545119 | 3.43642 | 0.000648 | 0.00126 | -1.58373 |
| PEA15 | 1.148847 | 11.4643 | 12.55002 | 6.08E-31 | 4.65E-29 | 59.4395 |
| LZTS1 | 1.178883 | 6.603971 | 6.508245 | 2.15E-10 | 1.06E-09 | 12.75688 |
| ATP6V1B1 | 1.221403 | 2.302243 | 4.668386 | 4.08E-06 | 1.13E-05 | 3.21165 |
| GBA | 1.264631 | 11.3375 | 11.94503 | 1.50E-28 | 8.62E-27 | 53.98056 |
| ZKSCAN3 | 1.349393 | 6.284392 | 10.17302 | 6.74E-22 | 1.35E-20 | 38.82135 |
| ITGA6 | 1.362832 | 10.41179 | 10.12482 | 1.00E-21 | 1.92E-20 | 38.42864 |
| NRG2 | 1.432955 | 2.914887 | 4.699698 | 3.53E-06 | 9.87E-06 | 3.350893 |
| UCHL1 | 1.432997 | 4.484096 | 3.557105 | 0.000417 | 0.000836 | -1.17414 |
| HSPB1 | 1.442621 | 13.43176 | 9.016675 | 6.73E-18 | 9.36E-17 | 29.72884 |
| DAPK2 | 1.477636 | 7.693333 | 8.025522 | 1.00E-14 | 7.92E-14 | 22.5345 |
| GPSM1 | 1.677399 | 7.599727 | 6.681859 | 7.44E-11 | 4.02E-10 | 13.7936 |
| TREM2 | 1.69756 | 6.162806 | 6.344954 | 5.72E-10 | 2.73E-09 | 11.8022 |
| ATP6V0D2 | 1.926682 | 2.96175 | 6.319992 | 6.63E-10 | 3.07E-09 | 11.65802 |
| TMEM150B | 2.33797 | 6.180484 | 7.729046 | 7.93E-14 | 5.87E-13 | 20.50099 |
| MAPT | 2.592561 | 6.553268 | 11.26366 | 6.33E-26 | 2.64E-24 | 47.99482 |
| TP73 | 2.837189 | 4.311532 | 10.40484 | 9.83E-23 | 2.65E-21 | 40.72585 |
| EEF1A2 | 3.183213 | 7.509015 | 5.176163 | 3.50E-07 | 1.10E-06 | 5.572082 |
| CDKN2A | 3.927211 | 7.272182 | 13.86813 | 2.50E-36 | 5.75E-34 | 71.73549 |
| BIRC5 | 4.310741 | 6.862221 | 16.67887 | 2.18E-48 | 1.00E-45 | 99.29609 |
